# Ultrasonic Disintegration to Improve Anaerobic Digestion of Microalgae with Hard Cell Walls—*Scenedesmus* sp. and *Pinnularia* sp.

**DOI:** 10.3390/plants12010053

**Published:** 2022-12-22

**Authors:** Marcin Dębowski, Joanna Kazimierowicz, Izabela Świca, Marcin Zieliński

**Affiliations:** 1 Department of Environmental Engineering, Faculty of Geoengineering, University of Warmia and Mazury in Olsztyn, 10-720 Olsztyn, Poland; 2 Department of Water Supply and Sewage Systems, Faculty of Civil Engineering and Environmental Sciences, Bialystok University of Technology, 15-351 Bialystok, Poland

**Keywords:** microalgae biomass, anaerobic digestion, ultrasound disintegration, biogas, methane, cell wall, pretreatment, biofuels

## Abstract

Microalgae are considered to be very promising feedstocks for biomethane production. It has been shown that the structure of microalgal cell walls can be highly detrimental to the anaerobic digestibility of biomass. Therefore, there is a real need to seek ways to eliminate this problem. The aim of the present study was to assess the effect of ultrasonic disintegration of *Scenedesmus* sp. and *Pinnularia* sp. microalgal biomass on the performance and energy efficiency of anaerobic digestion. The pretreatment was successful in significantly increasing dissolved COD and TOC in the system. The highest CH_4_ yields were noted for *Scenedesmus* sp. sonicated for 150 s and 200 s, which produced 309 ± 13 cm^3^/gVS and 313 ± 15 cm^3^/gVS, respectively. The 50 s group performed the best in terms of net energy efficiency at 1.909 ± 0.20 Wh/gVS. Considerably poorer performance was noted for *Pinnularia* sp., with biomass yields and net energy gains peaking at CH_4_ 250 ± 21 cm^3^/gVS and 0.943 ± 0.22 Wh/gVS, respectively. Notably, the latter value was inferior to even the non-pretreated biomass (which generated 1.394 ± 0.19 Wh/gVS).

## 1. Introduction

The current pollutant emission limits necessitate measures such as development and widespread deployment of renewable energy sources [1]. Development of biomass energy systems could do much to contribute to this goal [2,3]. Mismanagement of traditional energy crops can lead to a negative energy balance, reduced global food supply, and significant inflation of food prices. This necessitates the pursuit of competitive biomass sources [4,5]. From the standpoint of environmental policy, circular economy, and bioeconomy, the best strategy moving forward is to recycle and reuse municipal, agricultural, and industrial waste [6,7,8]. Another feasible alternative may be to harness microalgae, especially given their ability to rapidly build biomass, accumulate high-energy products in their cells, digest waste materials, and grow on land unsuitable for other purposes [9,10].

Microalgae-to-heat processes span a very wide range of methods: from simple combustion, through more advanced thermochemical methods (such as gasification and pyrolysis), to biodiesel generation and biotechnological ethanol/methane/biohydrogen harvesting [11]. Numerous studies have argued that anaerobic digestion (AD) is the most promising method for producing energy from algae, both in terms of performance and cost-effectiveness [12,13,14]. Algae-to-methane processes have been shown to be cost-effective and comparable to cellular lipid extraction in terms of harvested energy (in the form of biodiesel) [15]. In addition to high-energy biogas, the process also adds value in the form of digestate, which can be used directly as fertilizer or processed and reintroduced into the algal biomass culture as a medium component [16,17].

The performance of microalgal anaerobic digestion is limited by several factors, including the high nitrogen content of the biomass and the resultant low C/N [18]. The high amounts of microalgal protein lead to the formation of ammonia, which is toxic to fermentative bacteria [19]. Methanogenesis can also be inhibited by the sodium ions present in halophilous algae [20]. This can be successfully mitigated by co-digesting microalgal biomass with other organic feedstocks [21,22]. There have also been reports on adapting anaerobic bacterial communities to digest microalgal mono-feedstock [23,24].

The structure of the microalgae cell walls is more important in determining the specifics of the AD process than the characteristics of the biomass [25]. All species of microalgae that are readily biodegradable under anaerobic conditions have either no cell wall at all (e.g., *Dunaliella salina*) or possess a protein-based cell wall with no hard-to-degrade cellulose and hemicellulose components (e.g., *Chlamydomonas reinwardtii*, *Arthrospira platensis* and *Euglena gracilis*) [26]. Unlike these species, *Chlorella kessleri* and *Scenedesmus obliquus* do possess a hemicellulose cell wall [27]. In fact, the *Scenedesmus obliquus* cell wall has been singled out in the literature as particularly difficult to biodegrade due to its content of the biopolymer sporopollenin [28]. Even more complex is the silica cell wall of the *Bacillariophyceae* [29]. There are processing issues caused by the fact that microalgae such as the *Chlorella* sp. taxons—i.e., those that have well-explored cultivation processes, are quick to grow biomass, and are tolerant to contaminants in the medium—also happen to have biodegradation-resistant cell structures [30].

Therefore, there is a real need to seek methods that eliminate or significantly reduce those morphological quirks of microalgae, which limit AD performance [31]. Disintegration of complex organic feedstocks can be achieved with the various, well explored methods of pre-treatment, which provide benefits, such as better biodegradability, higher biomethane yields, and greater mineralization of the digestate [32]. This helps reduce digester size, space requirements, and costs, while also improving cost-effectiveness [33]. Ultrasonic treatment has been one of the methods used to this end [34]. This form of pre-treatment has been proven effective as a first round of processing before digestion of sludge [35], plant biomass [36], and dairy waste [37]. Ultrasonics have also been used to disinfect water [38], remove ammonia from wastewater [39], and support membrane filtration [40]. The term ‘ultrasound’ refers to soundwaves having a frequency above 16 kHz, the effects of which are separated into thermal and nonthermal [41]. Thermal effects occur when energy absorbed by matter is converted into heat, whereas nonthermal effects can be classified into cavitation and stress mechanisms [42]. A short retention time of the biomass in the ultrasound-exposed area is required [43]. This directly affects the small size of the equipment and the low demand for investment space [44]. The process is automated, fully monitored, and controlled [45]. The use of ultrasonic disintegration (UD) brings very good final results for substrates with hydration above 95% [46]. The disadvantages include high energy demand [47], significant operating costs of service, and periodic repairs, which mainly concern the erosion of probes and wear of ultrasonic heads [48], as well as the need to have qualified staff for effective use of the installation [49].

The choice of microalgae species tested in the experiments resulted from the fact that the methods of their cultivation are well known and allow to obtain a large amount of biomass [50,51]. Both species are resistant to adverse environmental conditions eurybionts and can be cultured using a variety of waste, including wastewater and digestate effluents [52,53]. In view of the above, these species are seen as a potential source of organic substrate for anaerobic digestion [54]. The technological problem concerns the presence of a cell structure resistant to biodegradation under anaerobic conditions [55]. Insufficient attention has been devoted to the intensification of methane fermentation of microalgae biomass by the use of ultrasonic disintegration, and the conducted research partially fills this gap.

The aim of the study was to determine the applicability of ultrasounds for the disintegration of *Scenedesmus* sp. and *Pinnularia* sp. microalgal biomass prior to anaerobic digestion (AD), as well as to assess the impact of this pre-treatment on biogas productivity and composition. We verified the effect of the ultrasound dose on the anaerobic degradation process, biogas production rate, and the potential for achieving net energy gains.

## 2. Results and Discussion

### 2.1. Changes in Dissolved Organics

The effectiveness of pre-treatment methods is often evaluated by monitoring the changes in dissolved organics levels [56]. Various metrics can be used, depending on the organic feedstock used, including levels of volatile solids, genetic material, lipids, glucose and biodegradable organic compounds (expressed as biochemical oxygen demand (BOD)) [57,58]. Chemical oxygen demand (COD) and total organic carbon (TOC) are some of the most universal and commonly used parameters [59]. The AD process and its performance are partly predicated on ensuring efficient transfer of organic substances from the biomass to the dissolved phase [60].

**Figure 1:**
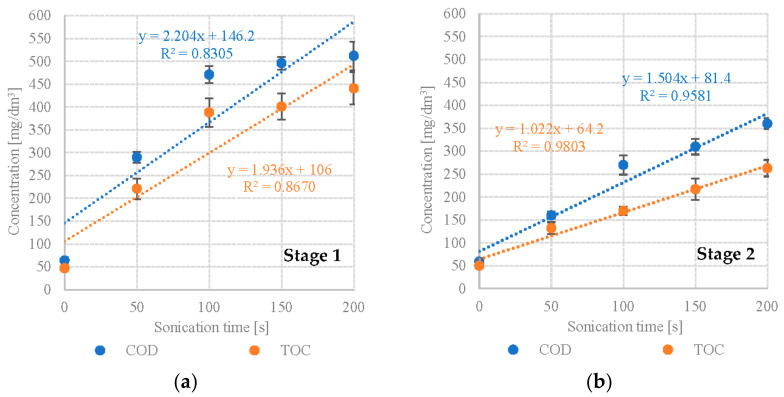
Correlations between UD exposure time and levels of dissolved organic compounds (**a**) S1—*Scenedesmus* sp., (**b**) S2—*Pinnularia* sp.

Our study showed a significant increase in the levels of monitored dissolved organic compounds. S1 showed significant increases in COD and TOC in variants S1V1 to S1V3. S1V1 (*Scenedesmus* sp., no DU) had a COD of 64 ± 7 mgO_2_/dm^3^ and TOC of 47 ± 6 mg/dm^3^ (Table 1). The values for S1V3 were 471 ± 19 mgO_2_/dm^3^ and 388 ± 31 mg/dm^3^, respectively (Table 1). The gains were less dramatic in the subsequent variants. In S1V5, the COD was 512 ± 31 mgO_2_/dm^3^, whereas TOC was 441 ± 35 mg/dm^3^ (Table 1). UD exposure time was found to strongly and positively correlate with dissolved COD (R^2^ = 0.8305) and TOC (R^2^ = 0.8670) levels (Figure 1a). S2 (*Pinnularia* sp.) experienced a consistent, statistically significant rise in levels of dissolved organic compounds across all of the tested UD intensities. COD increased from 59 ± 4 mgO_2_/dm^3^ in S2V1 to 360 ± 12 mgO_2_/dm^3^ in S2V5 (Table 1). TOC ranged from 50 ± 2 mg/dm^3^ to 263 ± 18 mg/dm^3^ (Table 1). The concentrations of dissolved organic compounds were much lower than in S1. UD exposure time in S2 was found to correlate very strongly and positively with dissolved COD (R^2^ = 0.9581) and TOC (R^2^ = 0.9803) levels (Figure 1b).

Cho et al. (2013) [61] have also found a significant increase in the levels of dissolved organic compounds after treating microalgal biomass (a mixture of Chlorella sp. and *Scenedesmus* sp.) with UD. The ultrasonic pretreatment was applied for 30, 90 and 180 s at 130 W. The initial soluble COD in the non-pretreated microalgal biomass was 770 mg/dm^3^, rising to 973 mg/dm^3^ after 30 s disintegration, 1639 mg/dm^3^ after 90 s, and 2282 mg/dm^3^ after 180 s [61]. Similarly, Gruber-Brunhumer et al. (2015) [62] demonstrated increases in soluble COD after applying ultrasound pretreatment to Acutodesmus obliquus. Ultrasonication led to 538.0 ± 59.6 g/kgTS, compared to the 383.5 ± 4.4 g/kgTS in the non-pretreated control [62]. Tensions appear in the biomass subjected to UD, which cause numerous changes in the cell structure. They are the effect of ultrasonic pressure, forces related to the change in viscosity, the phenomenon of moving a biological object in the medium and the increase in temperature [63]. UDs cause twisting, rotation or spinning of macromolecules with asymmetric shapes [64]. These phenomena cause a change in the charge of the cell surface, a change in the permeability of the cell membrane, rupture, disintegration and fragmentation of the cell membrane [65]. This allows organic compounds to transfer to the dissolved phase and thus be more available to anaerobic bacteria in the AD process [66].

### 2.2. Biogas and Methane Production

**Figure 2:**
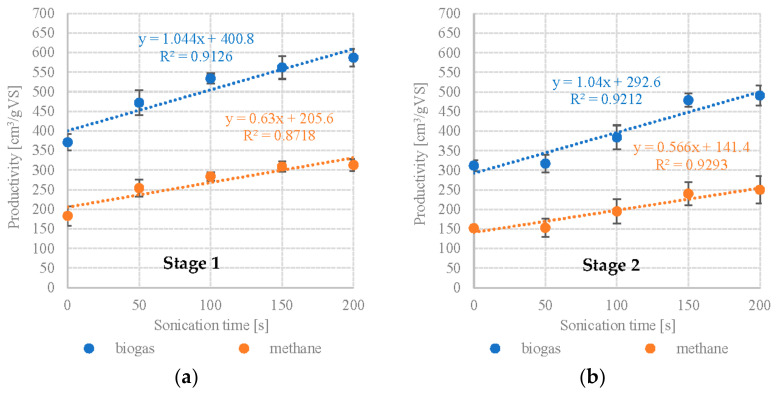
Correlations between UD exposure time and biogas/methane production (**a**) S1—*Scenedesmus* sp., (**b**) S2—*Pinnularia* sp.

The present study showed that UD had a positive effect on anaerobic digestion of microalgal biomass, providing significantly better biogas and methane production performance during digestion of *Scenedesmus* sp. Specific biogas yield in S1V1 was 371 ± 21 cm^3^/gVS with a CH_4_ fraction of 49.2 ± 2.4%, which corresponds to CH_4_ productivity of 183 ± 25 cm^3^/gVS. Significant improvements in anaerobic digestion performance were observed up to S1V3. Extending UD duration to 100 s boosted the CH_4_ yields to 284 ± 11 cm^3^/gVS. The highest AD performance for *Scenedesmus* sp. was obtained in S1V4 and S1V5 (150 s and 200 s UD, respectively), with no statistically significant differences in CH_4_ production between the two (S1V4—309 ± 13 cm^3^/gVS, S1V5—313 ± 15 cm^3^/gVS). The CH_4_ fraction in the biogas was similar across all of the UD variants, falling within the range of 53.2 ± 1.7% (S1V3) to 54.9 ± 0.9 (S1V4). A very strong positive correlation (R^2^ = 0.9126) and a strong positive correlation (R^2^ = 0.8718) were found between UD exposure time and biogas/methane production (Figure 2a).

On the other hand, 50 s UD applied to *Pinnularia* sp. did not significantly affect anaerobic digestion performance in terms of biogas yield and composition. S2V1 yielded 312 ± 14 cm^3^/gVS biogas containing 48.8 ± 3.0% CH_4_, whereas S2V2 produced 317 ± 23 cm^3^/gVS and 48.2 ± 2.1% CH_4_ (Table 2). Variant S2V3 and S2V4 showed incremental increases in biogas yield at 384 ± 31 cm^3^/gVS and 479 ± 17 cm^3^/gVS, respectively (Table 2). Increasing UD exposure time to 200 s produced no further significant gains in AD performance. The UD treatment also did not produce any statistically significant changes in the CH_4_ fraction in the biogas, which varied between 48.8 ± 3.0% in S2V1 to 50.9 ± 1.6% in S2V5 (Table 2). UD exposure time was found to strongly and positively correlate with biogas (R^2^ = 0.9212) and methane (R^2^ = 0.9293) production (Figure 2b).

Gruber-Brunhumer et al. (2015) [62] produced findings similar to our own, noting an increase in methane outputs from AD of *A. obliquus* from 191 m^3^/tCOD in the control to 292 m^3^/tCOD after UD, which translates to a 51% increase in methane yields. A positive effect of UD on microalgal anaerobic digestion performance was also demonstrated by Banu et al. (2020) [67], who optimized their UD experiment by screening different power levels (100 W–180 W) and different disintegration periods (0 min–120 min). Biogas production for the UD-treated mixed microalgal biomass peaked at 185.9 cm^3^/g COD biogas, whereas the non-pretreated control produced only 17 cm^3^/g COD [67]. In contrast, Cho et al. (2013) [61] obtained only slight uptick in methane yields after subjecting *Chlorella* sp. and *Scenedesmus* sp. to UD. After 30 s, 90 s and 180 s UD, the yields rose to 356 cm^3^/gVS, 368 cm^3^/gVS and 385 cm^3^/gVS methane, respectively (the non-disintegrated control produced 336 cm3/gVS) [61]. Caporgno et al. (2016) [68] also found that methane yields from *Phaeodactylum tricornutum* were unaffected by increasing UD energy levels. Energy inputs of 21 MJ/kgTS, 36 MJ/kgTS and 52 MJ/kgTS prior to AD resulted in yields of 287 ± 11 cm^3^/gVS, 284 ± 9 cm^3^/gVS and 285 ± 4 cm^3^/gVS, respectively—a mere 10% increase in methane production compared with non-treated microalgae (258 ± 12 cm^3^/gVS). The authors see this as further proof that the refractory nature of the organic fraction in *Phaeodactylum tricornutum* is the main obstacle for methane production since the pre-treatment destroys microalgae cells, but does not increase degradability [68]. Biomass composition is the most important determinant of methane production in anaerobic digestion. Methane yields from algal biomass correlate mainly with the cellular lipid content [69].

### 2.3. Energy Balance

**Figure 3:**
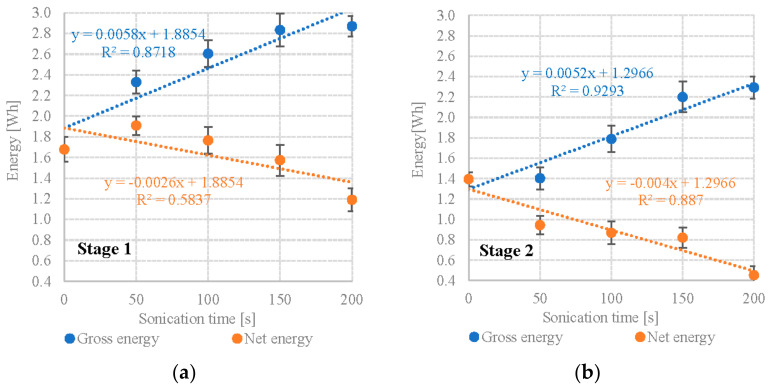
Correlations between UD exposure time and gross/net energy gain (**a**) S1—*Scenedesmus* sp., (**b**) S2—*Pinnularia* sp.

Calculating from the obtained methane yields and the calorific value of CH_4_ (9.17 Wh/dm^3^) shows that variants S1V4 and S1V5 performed the best in terms of gross energy production at 2.834 ± 0.12 Wh/gVS and 2.870 ± 0.14 Wh/gVS (Table 3). In contrast, S1V1 (no UD pretreatment) generated only 1.678 ± 0.23 Wh/gVS (Table 3). The energy consumed by the UD biomass pre-treatment was proportional to its duration, starting from 0.420 Wh/gVS and peaking at 1.680 Wh/gVS (Table 3). When factoring in the energy inputs, S1V2 and S1V3 are shown to have significantly higher net energy values: 1.909 ± 0.20 Wh/gVS and 1.764 ± 0.10 Wh/gVS, respectively (Table 3). The net energy gain for other variants was significantly lower than the control, varying between 1.574 ± 0.12 Wh/gVS and 1.190 Wh/gVS (Table 3). A strong positive correlation (R^2^ = 0.8718) was found between the DU exposure time and gross energy gain. Conversely, a moderate negative correlation (R^2^ = 0.5837) was found between the DU exposure time and net energy gain (Figure 3a). S2 (*Pinnularia* sp.) failed to produce a net energy balance in any of its UD variants, whereas the control showed a net energy gain of 1.394 ± 0.19 Wh/gVS, the UD variants yielded between 0.943 ± 0.22 Wh/gVS (S2V2) and 0.453 ± 0.21 Wh/gVS (S2V5) (Table 3). A very strong positive correlation (R^2^ = 0.9293) was found between the DU exposure time and gross energy gain. Conversely, a strong negative correlation (R^2^ = 0.8870) was found between the DU exposure time and net energy gain (Figure 3b). Studies have shown that a positive energy balance in relation to the control sample, where UD (V1) was not used, was obtained only when *Scenedesmus* sp. biomass was tested in S1V2 and S1V3. It was, respectively, 0.231 ± 0.02 Wh and 0.086 ± 0.07 Wh (Table 3). In the remaining variants, the net energy gain differential was negative. However, it must be emphasized that the results obtained on a laboratory scale can only be the initial basis for further research. More reliable data used for reliable LCA, LCC and in-depth energy and economic balance analysis can be obtained in tests conducted in conditions close to the real one. Pilot-scale studies reduce the differences and uncertainties associated with the scale-up process.

Energy efficiency is a major factor in viability assessments, especially for large-scale processes [70]. Cho et al. (2013) [61] investigated net energy balance as part of their study and found ultrasonic pre-treatment of microalgal biomass to be net energy negative. UD exposure times of 30 s, 90 s, and 180 s resulted in net energy production of −26.4 kJ/gVS, −104.0 kJ/gVS, and −220.4 kJ/gVS, respectively, calculated from energy inputs of 39 kJ/gVS, 117 kJ/gVS, and 234 kJ/gVS, respectively, and outputs of 12.6 kJ/gVS, 13.0 kJ/gVS, and 13.6 kJ/gVS, respectively [61]. Banu et al. (2020) [67] found that maximum solubilization of COD was achieved after applying −2542.55 kWh ultrasonic energy per ton biomass. The energy yield from the methane was calculated to be 333.38 kWh/ton biomass, meaning that the net energy balance was −2209.17 kWh/ton biomass [67]. Biomass density seems to be a key factor in determining the energy balance of the process. Due to the specific energy applied, which is inversely proportional to the initial solid concentration, it is possible to reach a positive energy balance by increasing the solids in the system. This means that the harvested microalgal biomass should not only be thickened, but also dewatered before ultrasound pre-treatment [71].

## 3. Materials and Methods

### 3.1. Experimental Design

This study on the effect of ultrasonic disintegration (UD) on the performance of microalgal biomass AD was separated into two stages, each focusing on a different species of microalgae: *Scenedesmus* sp. in stage 1 (S1) and *Pinnularia* sp. in stage 2 (S2). Each stage was subdivided into five experimental variants (V1–V5) with different rates of ultrasonic energy applied to the feedstock. The energy dose was adjusted by modifying the biomass retention time in the sonification zone. The experimental design, UD exposure time, and energy input values are presented in Table 4.

### 3.2. Materials

*Scenedesmus* sp. (UTEX 1589) and *Pinnularia* sp. (UTEX LB FD462)—S1 and S2, respectively—were grown in tap water at 20 ± 1 °C and illuminated with a 3500 lux fluorescent lamp under a 12 h light/12 h dark regime. Ambient air fed via a diffuser system (with a capacity of 150 dm^3^/h) served as the source of carbon dioxide. S1 used Bold’s basal medium (BBM) containing (dm^−1^): KH_2_PO_4_ (175 mg), CaCl_2_·2H_2_O (25 mg), MgSO_4_·7H_2_O (75 mg), NaNO_3_ (250 mg), K_2_HPO_4_ (75 mg), NaCl (25 mg), H_3_BO_3_ (11.42 mg), ZnSO_4_·7H_2_O (8.82 mg), MnCl_2_·4H_2_O (1.44 mg), MoO_3_ (0.71 mg), CuSO_4_·5H_2_O (1.57 mg), Co(NO_3_)_2_·6H_2_O (0.49 mg), Na_2_EDTA (50 mg), KOH (3.1 mg), FeSO_4_ (4.98 mg), and 1 µL of concentrated H_2_SO_4_. S2 used a modified combo medium containing (dm^−1^): NaNO_3_ (85.01 mg), CaCl_2_·2H_2_O (36.76 mg), MgSO_4_·7H_2_O (36.97 mg), NaHCO_3_ (12.6 mg), Na_2_SiO_3_·9H_2_O (28.42 mg), K_2_HPO_4_ (8.71 mg), H_3_BO_3_ (24 mg), KCl (7.45 mg), algal trace element solution (10 mL), vitamin B_12_ (1 mL), biotin (1 mL), and thiamine (1 mL).

The cultured microalgal biomass was separated using a vacuum membrane filtration kit (MDS 1, Whatman), which included a 50 mm MCE (mixed cellulose ester) filter insert (5.0 μm porosity). The microalgal biomass was separated from the culture medium in the filter compartment by applying 0.5 atm suction with a vacuum pump (Mobil 20). The membrane separation process produced thickened biomass containing an average 97 ± 1% water. The biomass profiles are given in Table 2. 500 cm^3^ of the thickened biomass was fed into the ultrasonic disintegrator then into the digestion respirometers. The anaerobic sludge inoculum was sourced from the digesters of the wastewater treatment plant in Olsztyn (Table 5). The digester operational parameters were: organic load rate 2.5 kg VS/m^3^·d, hydraulic retention time 20 d, and temperature 35 °C.

### 3.3. Experimental Set-Up

The microalgae biomass was subjected to UD with an UP 400S ultrasonic disintegrator (Hielscher Ultrasonics GmbH, Teltow, Germany) running at 400 W power and 24 kHz frequency. In successive variants, the ultrasound exposure time was incrementally increased while keeping the volume of feedstock constant (Table 4). After the UD, the anaerobic digestibility of the biomass was tested. Methane output was measured using the volumetric gas production method in batch respirometric reactors (AMPTS II, BPC Instruments AB, Lund, Sweden). The produced biogas was purified by an alkaline scrubbing solution (3M NaOH) to absorb CO_2_ and other non-methane gases in an ex situ absorption unit. The digestion process was run at 37 ± 1 °C. The bioreactors were equipped with a vertical stirrer operating for 30 s every 10 min at 100 rpm. The active volume of the respirometers was 200 cm^3^. Initial organic load rate (OLR) was 5.0 gVS/dm^3^. The quantities of feedstock injected into the respirometers are given in Table 6.

The ensure anaerobic conditions in the respirometers prior to the measurements, the system was purged with 150 dm^3^/h nitrogen for 5 min. Measurements were taken over a period of 40 d. A gas production report was software-logged once a day, using a program that generates results for a normalized gas volume (standard atmospheric pressure of 101.3 kPa at 0 °C and zero humidity). The readouts continued until the available organic compounds were completely decomposed. Successive biogas volume readouts were automatically compared against each other. The measurement was concluded when ten consecutive gas volume measurements were within 1% of each other. Endogenous biogas generated by anaerobic sludge was excluded from the calculation.

### 3.4. Analytical Measurements

Contents of dry matter, dry organic matter, and dry mineral matter were determined gravimetrically. Biomass samples desiccated at 105 °C were assayed for TC, TOC, and TN. The analysis was performed using a Thermo Flash 2000 organic elemental molecule analyzer (Thermo Scientific, Waltham, MA, USA). TP was determined colorimetrically in ammonium metavanadate (V) and ammonium molybdate after prior mineralization in a mixture of sulfuric (VI) and chloric (VII) acids at 390 nm using a DR 2800 spectrophotometer (Hach-Lange GmbH, Düsseldorf, Germany). Total protein was calculated by multiplying the value of TN by the protein conversion factor of 6.25. Reducing sugars were determined colorimetrically with an anthrone reagent at 600 nm using a DR 2800 spectrophotometer (Hach-Lange GmbH, Düsseldorf, Germany). Lipids were quantified using the Soxhlet method with a Büchi extraction apparatus (B-811, Büchi AG, Flawil, Switzerland). The pH determination procedure was as follows: 10 g of the homogenized air-dried sample was weighed out to a 100 mL beaker, after which 50 mL of distilled water was added, and sample pH was measured with a calibrated apparatus. The dissolved chemical oxygen demand (COD) was determined using a DR 5000 spectrophotometer with a HT 200 s mineralizer (Hach-Lange GmbH, Düsseldorf, Germany). Dissolved TOC was quantified by means of a TOC-L analyzer TOC-L (Shimadzu, Kyoto, Japan). Methane in the biogas was assayed with a GC Agillent 7890 A gas chromatograph (Santa Clara, CA, USA).

### 3.5. Calculation Methods

The specific energy input (E_in_) was calculated using Equation (1):

E_in_ = (P_D_⋅T_D_): M_VS_ [Wh/gVS] (1)

where: P_D_—disintegrator power [W], T_D_—disintegration time [h], and M_VS_—VS mass fed into the disintegrator [g].

The energy output (E_out_) generated from methane production was calculated using Equation (2):

E_out_ = Y_CH4_⋅CV_CH4_⋅M_VS_ [Wh/gVS] (2)

where: Y_CH4_—methane yield [dm^3^], CV_CH4_—methane calorific value [Wh/dm^3^], and M_VS_—VS mass injected into the respirometer [gVS].

The net energy gain (E_net_) was calculated using Equation (3):

E_net_ = E_out_ − E_in._ [Wh/gVS] (3)

### 3.6. Statistical Analysis

The experiments were conducted in four repetitions. The statistical analysis of experimental results was conducted using STATISTICA 13.1 PL (StatSoft, Inc., Tulsa, OK, USA). One-way analysis of variance (ANOVA) was used to determine differences between variables. Significant differences between the variables were determined via Tukey’s HSD. Results were considered significant at *p* = 0.05.

## 4. Conclusions

Pre-treating microalgal biomass with UD was found to significantly impact the performance of the anaerobic digestion (AD) process, directly affecting the levels of COD and TOC in the liquid phase. Dissolved organics significantly rose after *Scenedesmus* sp. was pretreated with UD, with a eight-fold increase for COD and almost a 9.5-fold increase for TOC. However, UD-treated *Pinnularia* sp. performed much worse.

The present study shows that UD has a positive effect on anaerobic digestion of microalgal biomass, providing significantly better performance with regard to *Scenedesmus* sp. digestion. The highest CH_4_ yields were noted for the 150 s and 200 s UD exposure times, as these variants produced 309 ± 13 cm^3^/gVS and 313 ± 15 cm^3^/gVS, respectively. The best performing *Pinnularia* sp. variant yielded 250 ± 21 cm^3^CH_4_/gVS.

The 50 s UD/*Scenedesmus* sp. group performed the best in terms of net energy gain at 1.909 ± 0.20 Wh/gVS. The energy efficiency of the AD process was not significantly improved by extending the disintegration time, with results similar to or lower than the control group. The digestion of UD-treated *Pinnularia* sp. failed to perform better than the non-pretreated biomass in terms of energy yield.

## Figures and Tables

**Table 1:** Indicators of dissolved organic compounds.

Stage	Variant	Period of Sonification [s]	COD_dissolved_ [mgO_2_/dm^3^]	TOC_dissolved_ [mg/dm^3^]
S1	V1	0	64 ± 7	47 ± 6
	V2	50	290 ± 12	221 ± 23
	V3	100	471 ± 19	388 ± 31
	V4	150	496 ± 14	401 ± 29
	V5	200	512 ± 31	441 ± 35
S2	V1	0	59 ± 4	50 ± 2
	V2	50	160 ± 8	132 ± 13
	V3	100	270 ± 21	170 ± 9
	V4	150	310 ± 17	217 ± 23
	V5	200	360 ± 12	263 ± 18

**Table 2:** Biogas and methane production.

Variant	Parameter	Unit	*Scenedesmus* sp.	*Pinnularia* sp.
V1	Biogas	cm^3^/gVS	371 ± 21	312 ± 14
	CH_4_	%	49.2 ± 2.4	48.8 ± 3.0
		cm^3^/gVS	183 ± 25	152 ± 21
V2	Biogas	cm^3^/gVS	472 ± 32	317 ± 23
	CH_4_	%	53.9 ± 1.4	48.2 ± 2.1
		cm^3^/gVS	254 ± 22	153 ± 24
V3	Biogas	cm^3^/gVS	534 ± 13	384 ± 31
	CH_4_	%	53.2 ± 1.7	50.7 ± 1.3
		cm^3^/gVS	284 ± 11	195 ± 20
V4	Biogas	cm^3^/gVS	562 ± 29	479 ± 17
	CH_4_	%	54.9 ± 0.9	50.2 ± 2.7
		cm^3^/gVS	309 ± 13	240 ± 23
V5	Biogas	cm^3^/gVS	587 ± 22	491 ± 26
	CH_4_	%	53.3 ± 1.4	50.9 ± 1.6
		cm^3^/gVS	313 ± 15	250 ± 21

**Table 3:** Energy efficiency.

Stage	Variant	Reactor Active Volume [cm^3^]	OLR [gVS/dm^3^]	VS Fed into the Reactor, by Mass [g]	Specific Methane Output [cm^3^/gVS]	Specific Methane Energy Density [Wh/dm^3^]	Methane Energy Density [Wh/gVS]	Energy Consumed by Disintegration [Wh/g VS]	Net Energy Gain [Wh]	Net Energy Gain Differential V(x)-V1 [Wh] *
S1	V1	200	5	1	183 ± 25	9.17	1.678 ± 0.23	0	1.678 ± 0.23	-
	V2				254 ± 22		2.329 ± 0.20	0.420	1.909 ± 0.20	0.231 ± 0.02
	V3				284 ± 11		2.604 ± 0.10	0.840	1.764 ± 0.10	0.086 ± 0.07
	V4				309 ± 13		2.834 ± 0.12	1.260	1.574 ± 0.12	−0.104 ± 0.04
	V5				313 ± 15		2.870 ± 0.14	1.680	1.190 ± 0.14	−0.488 ± 0.08
S2	V1				152 ± 21		1.394 ± 0.19	0	1.394 ± 0.19	-
	V2				153 ± 24		1.403 ± 0.22	0.460	0.943 ± 0.22	−0.451 ± 0.02
	V3				195 ± 20		1.788 ± 0.18	0.920	0.868 ± 0.18	−0.526 ± 0.05
	V4				240 ± 21		2.201 ± 0.19	1.380	0.821 ± 0.19	−0.573 ± 0.06
	V5				250 ± 23		2.293 ± 0.21	1.840	0.453 ± 0.21	−0.941 ± 0.07

* Net Energy gain differential is the difference between net energy gain [Wh] obtained in subsequent variants (V2, …, V5) and net energy gain [Wh] obtained in V1 (without using ultrasonic disintegration).

**Table 4:** Experimental design.

Stage	Variant	Sonification Period [s]	Energy Input [Wh]	Volume [cm^3^]	Dry Mass [g]	Volatile Solids [g]	Energy Input [Wh/gVS]
S1 *Scenedesmus* sp.	V1	0	-	500	15	13.2	0
	V2	50	5.55				0.42
	V3	100	11.10				0.84
	V4	150	16.65				1.26
	V5	200	22.20				1.68
S2 *Pinnularia* sp.	V1	0	-			12.0	0
	V2	50	5.55				0.46
	V3	100	11.10				0.92
	V4	150	16.65				1.38
	V5	200	22.20				1.84

**Table 5:** Profile of the microalgal biomass and anaerobic sludge.

Parameter	Unit	Value		
		*Scenedesmus* sp.	*Pinnularia* sp.	Anaerobic Sludge
Organic dry mass (VS)	[%TS]	88.4 ± 0.7	79.9 ± 1.6	71.2 ± 3.1
Mineral dry mass (MS)	[%TS]	11.6 ± 0.7	20.1 ± 1.6	28.8 ± 2.0
Total nitrogen (TN)	[mg/gTS]	44.1 ± 1.4	42.9 ± 1.1	33.8 ± 5.2
Total phosphorus (TP)	[mg/gTS]	17.8 ± 1.1	12.7 ± 0.5	2.0 ± 0.3
Total carbon (TC)	[mg/gTS]	511 ± 12	409 ± 31	705.8 ± 19.4
Total organic carbon (TOC)	[mg/gTS]	452 ± 27	357 ± 19	577.8 ± 21.8
C:N ratio	-	10.3 ± 0.8	8.31 ± 0.5	17.1 ± 0.2
pH	-	7.52 ± 0.13	7.61 ± 0.09	7.23 ± 0.11
Protein	[%TS]	27.5 ± 0.6	26.8 ± 0.5	21.1 ± 0.3
Lipids	[%TS]	16.3 ± 0.90	9.4 ± 2.1	4.1 ± 1.3
Saccharides	[%TS]	36.8 ± 2.2	35.3 ± 2.6	1.7 ± 0.5

**Table 6:** Quantities and volumes of microalgal biomass injected into the respirometers.

Stage	OLR	Respirometer V	Required VS	Biomass Water Content	TS in the Biomass	VS	VS in the Biomass	V of Respirometer Input (Biomass)
	gVS/dm^3^	cm^3^	g	%	g/dm^3^	%TS	g/dm^3^	cm^3^
S1—*Scenedesmus* sp.	5.0	200	1.0	97	30	88.4	26.5	37.7
S2—*Pinnularia* sp.						79.9	24.0	41.7

## Data Availability

Not applicable.

## References

[1] Ilechukwu N., Lahiri S. (2022). Renewable-Energy Consumption and International Trade. Energy Rep..

[2] Vujanović M., Wang Q., Mohsen M., Duić N., Yan J. (2021). Recent Progress in Sustainable Energy-Efficient Technologies and Environmental Impacts on Energy Systems. Appl. Energy.

[3] Kazimierowicz J. (2014). Organic Waste Used In Agricultural Biogas Plants. J. Ecol. Eng..

[4] Dębowski M., Kazimierowicz J., Zieliński M., Bartkowska I. (2022). Co-Fermentation of Microalgae Biomass and *Miscanthus × Giganteus* Silage—Assessment of the Substrate, Biogas Production and Digestate Characteristics. Appl. Sci..

[5] Thanigaivel S., Priya A.K., Dutta K., Rajendran S., Vasseghian Y. (2022). Engineering Strategies and Opportunities of next Generation Biofuel from Microalgae: A Perspective Review on the Potential Bioenergy Feedstock. Fuel.

[6] Gil A. (2022). Challenges on Waste-to-Energy for the Valorization of Industrial Wastes: Electricity, Heat and Cold, Bioliquids and Biofuels. Environ. Nanotechnol. Monit. Manag..

[7] Kazimierowicz J., Dzienis L., Dębowski M., Zieliński M. (2021). Optimisation of Methane Fermentation as a Valorisation Method for Food Waste Products. Biomass Bioenergy.

[8] Vyas S., Prajapati P., Shah A.V., Kumar Srivastava V., Varjani S. (2022). Opportunities and Knowledge Gaps in Biochemical Interventions for Mining of Resources from Solid Waste: A Special Focus on Anaerobic Digestion. Fuel.

[9] Dębowski M., Dudek M., Zieliński M., Nowicka A., Kazimierowicz J. (2021). Microalgal Hydrogen Production in Relation to Other Biomass-Based Technologies—A Review. Energies.

[10] Dębowski M., Zieliński M., Krzemieniewski M., Dudek M., Grala A. (2012). Microalgae–Cultivation Methods. Pol. J. Nat. Sci..

[11] Yap J.K., Sankaran R., Chew K.W., Halimatul Munawaroh H.S., Ho S.H., Rajesh Banu J., Show P.L. (2021). Advancement of Green Technologies: A Comprehensive Review on the Potential Application of Microalgae Biomass. Chemosphere.

[12] Dębowski M., Zieliński M., Świca I., Kazimierowicz J. (2021). Algae Biomass as a Potential Source of Liquid Fuels. Phycology.

[13] Martín Juárez J., Riol Pastor E., Fernández Sevilla J.M., Muñoz Torre R., García-Encina P.A., Bolado Rodríguez S. (2018). Effect of Pretreatments on Biogas Production from Microalgae Biomass Grown in Pig Manure Treatment Plants. Bioresour. Technol..

[14] Klassen V., Blifernez-Klassen O., Bax J., Kruse O. (2020). Wastewater-Borne Microalga *Chlamydomonas* Sp.: A Robust Chassis for Efficient Biomass and Biomethane Production Applying Low-N Cultivation Strategy. Bioresour. Technol..

[15] Dębowski M., Zieliński M., Kisielewska M., Kazimierowicz J., Dudek M., Świca I., Rudnicka A. (2020). The Cultivation of Lipid-Rich Microalgae Biomass as Anaerobic Digestate Valorization Technology—A Pilot-Scale Study. Processes.

[16] Behera B., Selvam S.M., Paramasivan B. (2022). Research Trends and Market Opportunities of Microalgal Biorefinery Technologies from Circular Bioeconomy Perspectives. Bioresour. Technol..

[17] Razzak S.A., Lucky R.A., Hossain M.M., deLasa H. (2022). Valorization of Microalgae Biomass to Biofuel Production: A Review. Energy Nexus.

[18] Zabed H.M., Akter S., Yun J., Zhang G., Zhang Y., Qi X. (2020). Biogas from Microalgae: Technologies, Challenges and Opportunities. Renew. Sustain. Energy Rev..

[19] Veerabadhran M., Gnanasekaran D., Wei J., Yang F. (2021). Anaerobic Digestion of Microalgal Biomass for Bioenergy Production, Removal of Nutrients and Microcystin: Current Status. J. Appl. Microbiol..

[20] Tawfik A., Ismail S., Elsayed M., Qyyum M.A., Rehan M. (2022). Sustainable Microalgal Biomass Valorization to Bioenergy: Key Challenges and Future Perspectives. Chemosphere.

[21] Dȩbowski M., Kisielewska M., Kazimierowicz J., Rudnicka A., Dudek M., Romanowska-Duda Z., Zielínski M. (2020). The Effects of Microalgae Biomass Co-Substrate on Biogas Production from the Common Agricultural Biogas Plants Feedstock. Energies.

[22] Yin Y., Chen Y., Wang J. (2021). Co-Fermentation of Sewage Sludge and Algae and Fe2+ Addition for Enhancing Hydrogen Production. Int. J. Hydrogen Energy.

[23] Doloman A., Soboh Y., Walters A.J., Sims R.C., Miller C.D. (2017). Qualitative Analysis of Microbial Dynamics during Anaerobic Digestion of Microalgal Biomass in a UASB Reactor. Int. J. Microbiol..

[24] Klassen V., Blifernez-Klassen O., Wibberg D., Winkler A., Kalinowski J., Posten C., Kruse O. (2017). Highly Efficient Methane Generation from Untreated Microalgae Biomass. Biotechnol. Biofuels.

[25] Fernández-Rodríguez M.J., de la Lama-Calvente D., Jiménez-Rodríguez A., Borja R., Rincón-Llorente B. (2019). Influence of the Cell Wall of *Chlamydomonas Reinhardtii* on Anaerobic Digestion Yield and on Its Anaerobic Co-Digestion with a Carbon-Rich Substrate. Process Saf. Environ. Prot..

[26] Griffiths G., Hossain A.K., Sharma V., Duraisamy G. (2021). Key Targets for Improving Algal Biofuel Production. Clean Technol..

[27] Choi H.I., Sung Y.J., Hong M.E., Han J., Min B.K., Sim S.J. (2022). Reconsidering the Potential of Direct Microalgal Biomass Utilization as End-Products: A Review. Renew. Sustain. Energy Rev..

[28] Jothibasu K., Muniraj I., Jayakumar T., Ray B., Dhar D.W., Karthikeyan S., Rakesh S. (2022). Impact of Microalgal Cell Wall Biology on Downstream Processing and Nutrient Removal for Fuels and Value-Added Products. Biochem. Eng. J..

[29] Fischer H., Robl I., Sumper M., Kröger N. (1999). Targeting and covalent modification of cell wall and membrane proteins heterologously expressed in the diatom *Cylindrotheca Fusiformis* (bacillariophyceae). J. Phycol..

[30] Zhen G., Lu X., Kobayashi T., Kumar G., Xu K. (2016). Anaerobic Co-Digestion on Improving Methane Production from Mixed Microalgae (*Scenedesmus* Sp., *Chlorella* Sp.) and Food Waste: Kinetic Modeling and Synergistic Impact Evaluation. Chem. Eng. J..

[31] Mathushika J., Gomes C. (2022). Development of Microalgae-Based Biofuels as a Viable Green Energy Source: Challenges and Future Perspectives. Biointerface Res. Appl. Chem..

[32] Kazimierowicz J., Bartkowska I., Walery M. (2020). Effect of Low-Temperature Conditioning of Excess Dairy Sewage Sludge with the Use of Solidified Carbon Dioxide on the Efficiency of Methane Fermentation. Energies.

[33] Zielinski M., Debowski M., Kazimierowicz J. (2021). The Effect of Static Magnetic Field on Methanogenesis in the Anaerobic Digestion of Municipal Sewage Sludge. Energies.

[34] Rokicka M., Zieliński M., Dudek M., Dębowski M. (2021). Effects of Ultrasonic and Microwave Pretreatment on Lipid Extraction of Microalgae and Methane Production from the Residual Extracted Biomass. Bioenergy Res..

[35] Zawieja I., Włodarczyk R., Kowalczyk M. (2019). Biogas Generation from Sonicated Excess Sludge. Water.

[36] Kisielewska M., Rusanowska P., Dudek M., Nowicka A., Krzywik A., Dębowski M., Joanna K., Zieliński M. (2020). Evaluation of Ultrasound Pretreatment for Enhanced Anaerobic Digestion of *Sida Hermaphrodita*. Bioenergy Res..

[37] Kazimierowicz J., Zieliński M., Bartkowska I., Dębowski M. (2022). Effect of Acid Whey Pretreatment Using Ultrasonic Disintegration on the Removal of Organic Compounds and Anaerobic Digestion Efficiency. Int. J. Environ. Res. Public Health.

[38] Blume T., Neis U. (2004). Improved Wastewater Disinfection by Ultrasonic Pre-Treatment. Ultrason. Sonochem..

[39] Matouq M.A.D., Al-Anber Z.A. (2007). The Application of High Frequency Ultrasound Waves to Remove Ammonia from Simulated Industrial Wastewater. Ultrason. Sonochem..

[40] Kyllönen H., Pirkonen P., Nyström M., Nuortila-Jokinen J., Grönroos A. (2006). Experimental Aspects of Ultrasonically Enhanced Cross-Flow Membrane Filtration of Industrial Wastewater. Ultrason. Sonochem..

[41] Draye M., Estager J., Kardos N. (2019). Organic Sonochemistry: Ultrasound in Green Organic Synthesis. Act. Methods.

[42] Neumann P., Pesante S., Venegas M., Vidal G. (2016). Developments in Pre-Treatment Methods to Improve Anaerobic Digestion of Sewage Sludge. Rev. Environ. Sci. Bio/Technol..

[43] Kashyap N., Roy K., Moholkar V.S. (2020). Mechanistic Investigations in Ultrasound-Assisted Biodegradation of Phenanthrene. Ultrason. Sonochem..

[44] Lin Y., Feng L., Li X., Chen Y., Yin G., Zhou W. (2020). Study on Ultrasound-Assisted Oxidative Desulfurization for Crude Oil. Ultrason. Sonochem..

[45] Lippert T., Bandelin J., Vogl D., Tesieh Z.A., Wild T., Drewes J.E., Koch K. (2020). Full-Scale Assessment of Ultrasonic Sewage Sludge Pretreatment Using a Novel Double-Tube Reactor. ACS ES&T Eng..

[46] Atelge M.R., Atabani A.E., Banu J.R., Krisa D., Kaya M., Eskicioglu C., Kumar G., Lee C., Yildiz Y., Unalan S. (2020). A Critical Review of Pretreatment Technologies to Enhance Anaerobic Digestion and Energy Recovery. Fuel.

[47] Wen C., Zhang J., Zhang H., Dzah C.S., Zandile M., Duan Y., Ma H., Luo X. (2018). Advances in Ultrasound Assisted Extraction of Bioactive Compounds from Cash Crops—A Review. Ultrason. Sonochem..

[48] Salakkam A., Sittijunda S., Mamimin C., Phanduang O., Reungsang A. (2021). Valorization of Microalgal Biomass for Biohydrogen Generation: A Review. Bioresour. Technol..

[49] Deivayanai V.C., Yaashikaa P.R., Senthil Kumar P., Rangasamy G. (2022). A Comprehensive Review on the Biological Conversion of Lignocellulosic Biomass into Hydrogen: Pretreatment Strategy, Technology Advances and Perspectives. Bioresour. Technol..

[50] Lim J.H.K., Gan Y.Y., Ong H.C., Lau B.F., Chen W.H., Chong C.T., Ling T.C., Klemeš J.J. (2021). Utilization of Microalgae for Bio-Jet Fuel Production in the Aviation Sector: Challenges and Perspective. Renew. Sustain. Energy Rev..

[51] Putri E.S.K., Verawaty M. (2020). Microbial Community in Constructed Wetland during the Treatment of Domestic Wastewater. J. Phys. Conf. Ser..

[52] Ferreira A., Reis A., Vidovic S., Vladic J., Gkelis S., Melkonyan L., Avetisova G., Congestri R., Acién G., Muñoz R. (2019). Combining Microalgae-Based Wastewater Treatment with Biofuel and Bio-Based Production in the Frame of a Biorefinery. Grand Challenges in Algae Biotechnology.

[53] Zainith S., Saxena G., Kishor R., Bharagava R.N. (2021). Application of Microalgae in Industrial Effluent Treatment, Contaminants Removal, and Biodiesel Production: Opportunities, Challenges, and Future Prospects. Bioremediation for Environmental Sustainability, Toxicity, Mechanisms of Contaminants Degradation, Detoxification, and Challenges.

[54] Bhatt P., Bhandari G., Turco R.F., Aminikhoei Z., Bhatt K., Simsek H. (2022). Algae in Wastewater Treatment, Mechanism, and Application of Biomass for Production of Value-Added Product. Environ. Pollut..

[55] Khan M.J., Harish, Ahirwar A., Schoefs B., Pugazhendhi A., Varjani S., Rajendran K., Bhatia S.K., Saratale G.D., Saratale R.G. (2021). Insights into Diatom Microalgal Farming for Treatment of Wastewater and Pretreatment of Algal Cells by Ultrasonication for Value Creation. Environ. Res..

[56] Volschan Junior I., de Almeida R., Cammarota M.C. (2021). A Review of Sludge Pretreatment Methods and Co-Digestion to Boost Biogas Production and Energy Self-Sufficiency in Wastewater Treatment Plants. J. Water Process Eng..

[57] Lin Q., Dong X., Luo J., Zeng Q., Ma J., Wang Z., Chen G., Guo G. (2022). Electrochemical Pretreatment Enhancing Co-Fermentation of Waste Activated Sludge and Food Waste into Volatile Fatty Acids: Performance, Microbial Community Dynamics and Metabolism. Bioresour. Technol..

[58] Zamri M.F.M.A., Hasmady S., Akhiar A., Ideris F., Shamsuddin A.H., Mofijur M., Fattah I.M.R., Mahlia T.M.I. (2021). A Comprehensive Review on Anaerobic Digestion of Organic Fraction of Municipal Solid Waste. Renew. Sustain. Energy Rev..

[59] Sari Erkan H., Bakaraki Turan N. (2022). Effects of Hydrogen Peroxide and Calcium Hypochlorite on Chemical Oxygen Demand Solubilization and Disintegration of Waste Activated Sludge by Electro-Chemical Pretreatment. Environ. Technol..

[60] Lee J., Park K.Y. (2020). Impact of Hydrothermal Pretreatment on Anaerobic Digestion Efficiency for Lignocellulosic Biomass: Influence of Pretreatment Temperature on the Formation of Biomass-Degrading Byproducts. Chemosphere.

[61] Cho S., Park S., Seon J., Yu J., Lee T. (2013). Evaluation of Thermal, Ultrasonic and Alkali Pretreatments on Mixed-Microalgal Biomass to Enhance Anaerobic Methane Production. Bioresour. Technol..

[62] Gruber-Brunhumer M.R., Jerney J., Zohar E., Nussbaumer M., Hieger C., Bochmann G., Schagerl M., Obbard J.P., Fuchs W., Drosg B. (2015). *Acutodesmus Obliquus* as a Benchmark Strain for Evaluating Methane Production from Microalgae: Influence of Different Storage and Pretreatment Methods on Biogas Yield. Algal Res..

[63] Shanthi M., Sundaramahalingam M.A., Rajeshbanu J., Sivashanmugam P. (2023). Surfactant-Assisted Ultrasonic Fragmentation of Mixed Fruit and Vegetable Biomass: Its Impact on Biomethane Yield and Energy Analysis. Fuel.

[64] Teo H.L., Wahab R.A. (2020). Towards an Eco-Friendly Deconstruction of Agro-Industrial Biomass and Preparation of Renewable Cellulose Nanomaterials: A Review. Int. J. Biol. Macromol..

[65] Godvin Sharmila V., Kumar G., Sivashanmugham P., Piechota G., Park J.H., Adish Kumar S., Rajesh Banu J. (2022). Phase Separated Pretreatment Strategies for Enhanced Waste Activated Sludge Disintegration in Anaerobic Digestion: An Outlook and Recent Trends. Bioresour. Technol..

[66] Ouahabi Y.R., Bensadok K., Ouahabi A. (2021). Optimization of the Biomethane Production Process by Anaerobic Digestion of Wheat Straw Using Chemical Pretreatments Coupled with Ultrasonic Disintegration. Sustainability.

[67] Rajesh Banu J., Yukesh Kannah R., Kavitha S., Ashikvivek A., Bhosale R.R., Kumar G. (2020). Cost Effective Biomethanation via Surfactant Coupled Ultrasonic Liquefaction of Mixed Microalgal Biomass Harvested from Open Raceway Pond. Bioresour. Technol..

[68] Caporgno M.P., Olkiewicz M., Torras C., Salvadó J., Clavero E., Bengoa C. (2016). Effect of Pre-Treatments on the Production of Biofuels from *Phaeodactylum Tricornutum*. J. Environ. Manag..

[69] Zhao B., Ma J., Zhao Q., Laurens L., Jarvis E., Chen S., Frear C. (2014). Efficient Anaerobic Digestion of Whole Microalgae and Lipid-Extracted Microalgae Residues for Methane Energy Production. Bioresour. Technol..

[70] Passos F., García J., Ferrer I. (2013). Impact of Low Temperature Pretreatment on the Anaerobic Digestion of Microalgal Biomass. Bioresour. Technol..

[71] Rodriguez C., Alaswad A., Mooney J., Prescott T., Olabi A.G. (2015). Pre-Treatment Techniques Used for Anaerobic Digestion of Algae. Fuel Process. Technol..

